# Investigations on spreading of PRRSV among swine herds by improved minimum spanning network analysis

**DOI:** 10.1038/s41598-020-75516-5

**Published:** 2020-11-05

**Authors:** Péter Márton Szabó, Dóra Szalay, Sándor Kecskeméti, Tamás Molnár, István Szabó, Ádám Bálint

**Affiliations:** 1 Hungarian Academy of Sciences and Semmelweis University, Szigony u. 43., Budapest, 1083 Hungary; 2 Department of Virology, National Food Chain Safety Office Veterinary Diagnostic Directorate, Tabornok u. 2., Budapest, 1143 Hungary; 3 National PRRS Eradication Committee, Keleti Karoly u. 24., Budapest, 1024 Hungary

**Keywords:** Computational biology and bioinformatics, Evolution, Molecular biology, Diseases

## Abstract

In Hungary, the economic losses caused by porcine reproductive and respiratory syndrome virus (PRRSV) led to the launching of a national PRRSV Eradication Program. An important element of the program was investigating the spread of PRRSV among swine herds and the possible ways of introduction by sequencing of the open reading frame 5 (ORF5) gene. However, the classical phylogenetic tree presentation cannot explain several genetic relationships clearly, while more precise visualization can be represented by network tree diagram. In this paper, we describe a practical and easy-to-follow enriched minimum spanning similarity network application for improved representation of phylogenetic relations among viral strains. This method eliminated the necessity of applying a predefined, arbitrary cut-off or computationally extensive algorithms. The network-based visualization allowed processing and visualizing large amount of data equally for the laboratory, private and official veterinarians, and helped identify the potential connections between different viral sequences that support data-driven decisions in the eradication program. By applying network analysis, previously unknown epidemiological connections between infected herds were identified, and virus spreading was analyzed within short period of time. In our study, we successfully built and applied network analysis tools in the course of the Hungarian PRRSV Eradication Program.

## Introduction

Porcine reproductive and respiratory syndrome (PRRS) is a major infectious disease that causes severe losses in modern intensive swine production worldwide^1^. The disease is caused by an RNA virus (PRRS virus, PRRSV) belonging to the Porarterivirus genus. The clinical signs of the disease include reduction in reproductive performance of the sows and respiratory diseases in young piglets. The immunosuppressive effect of PRRSV in combination with other pathogens within the herd significantly increases morbidity and mortality^2^.

Eradication of PRRSV was carried out at national and regional levels in Chile^3^. At state level (Minnesota) in the USA, a relatively successful elimination program was implemented, but complete eradication was not achieved^4^. Among the EU Member States, a pilot program started in the Netherlands with local motivation of breeders. In Denmark, a voluntary monitoring program is expected in the near future^5^. A successful regional control program was reported which aimed to eliminate PRRSV from all herds on the Horne Peninsula, Denmark^6^. Czech authors reported successful elimination of PRRSV from an infected farrow-to-finish herd by vaccination^7^.

In Hungary, the prevalence of PRRSV infection and the economic losses led to the launching of a national PRRSV Eradication Program in 2014 that was based on territorial principles and was accepted by the EU competent veterinary health committee. As a result of the implemented program introduced in 2014, swine population of 10 out of 19 counties of Hungary became officially free from PRRSV by the end of 2018. To the authors’ knowledge, this is the largest successful European PRRSV area elimination project^8–10^.

In order to successfully implement any eradication program, special attention has to be paid to investigating the spread of PRRSV among swine herds and the possible ways in which farms can become positive^11^. This not only plays a significant role in this phase of the eradication, but also helps enhancing the biosecurity of the herds, thereby reducing the possibility of reinfection.

Regular serological and virological (PCR) laboratory monitoring of swine are relevant features of the Hungarian PRRSV eradication process. Sequencing the open reading frame 5 (ORF5) gene detected in the laboratory tests is an appropriate method for classifying different strains of PRRSV^5,12–15^. Similarity and phylogenetic relations between the individual virus strains can be represented by traditional phylogenetic trees. These data contribute to the estimation of the genetic distance between PRRSV strains detected in different swine herds, and subsequent epidemiological investigations can be initiated that can provide data about the source of infection^16^. However, exact epidemiological conclusions can be drawn if sampling is intense and well documented. The official sampling regime specified by the nationwide PRRSV Eradication Program in Hungary provided a possibility to fulfill these requirements.

The classical bifurcating phylogenetic representation of genetic relationship often fails to describe PRRSV transmission between herds if the size or incongruence of the data is great^17^. Moreover, patterns of the applied dataset that are well supported by the epidemiological data frequently do not appear clearly in the optimized phylogenetic tree^18^. In these cases, more precise visualization can be achieved by network tree diagrams^19^. Among them, minimum spanning network trees (MSTs) are very popular common tools for molecular epidemiology investigations. The members of a sequence set are compared pairwise in order to calculate the distances that give the level of difference among the respective sequences. MST visualizes a set of edges (distance relations) that connect together nodes (individual sequences) in the shortest distance. This path is considered as the most feasible chain of infection. However, the generated MST should be interpreted with caution, and complex analysis of the MST together with the epidemiological data is inevitable, since genetic similarity cannot always be correlated with direct transmission^20^. Vaccine derived viruses or PRRSVs imported from the same source to herds with different geographical locations can be close to one another, while convergent evolution of not distantly related viruses can also occur. Several other factors can also influence virus evolution, such as vaccination pressure, immune status of the host and transmission rate between farms.

Although a single MST is expected using several algorithms, frequently equally parsimonious paths are obtained when two or more edges have the same length. In this case, the most common solution is arbitrarily selection of one of the possible shortest paths for tree construction^21^. Most commercially available network software use this arbitrary cut-off value for distances (weights) of the edges to determinate which sequences are most likely related based on genomic similarities and for the network representation.

The identification of an ideal cut-off value is often challenging. If the selected value is too high, multiple disconnected nodes and networks would be generated. If the value is too low, the network could be heavily connected in certain areas and hard to visualize the highly important connections. Therefore, neither classical phylogenetic tree, nor network visualization allow full network coverage, and both methods make it difficult to provide a sufficiently clear representation of huge number of sequences detected during the eradication process.

The aim of the present study was to develop an improved (extended) MST with optimized cut-off values that has strong correlation with the official clades^15^ and lineages^16^ obtained by classical phylogeny. Representing the optimally enriched number of connections of a particular sequence, network characteristics (degree, centrality, modules, etc.) may reveal important epidemiological information that is not present in the classical phylogenetic tree. These data can be used by the central and the local veterinary authority for epidemic investigation in order to determine the most likely source of PRRSV transmission as soon as possible.

## Results

### Phylogenetic analysis

**Figure 1:**
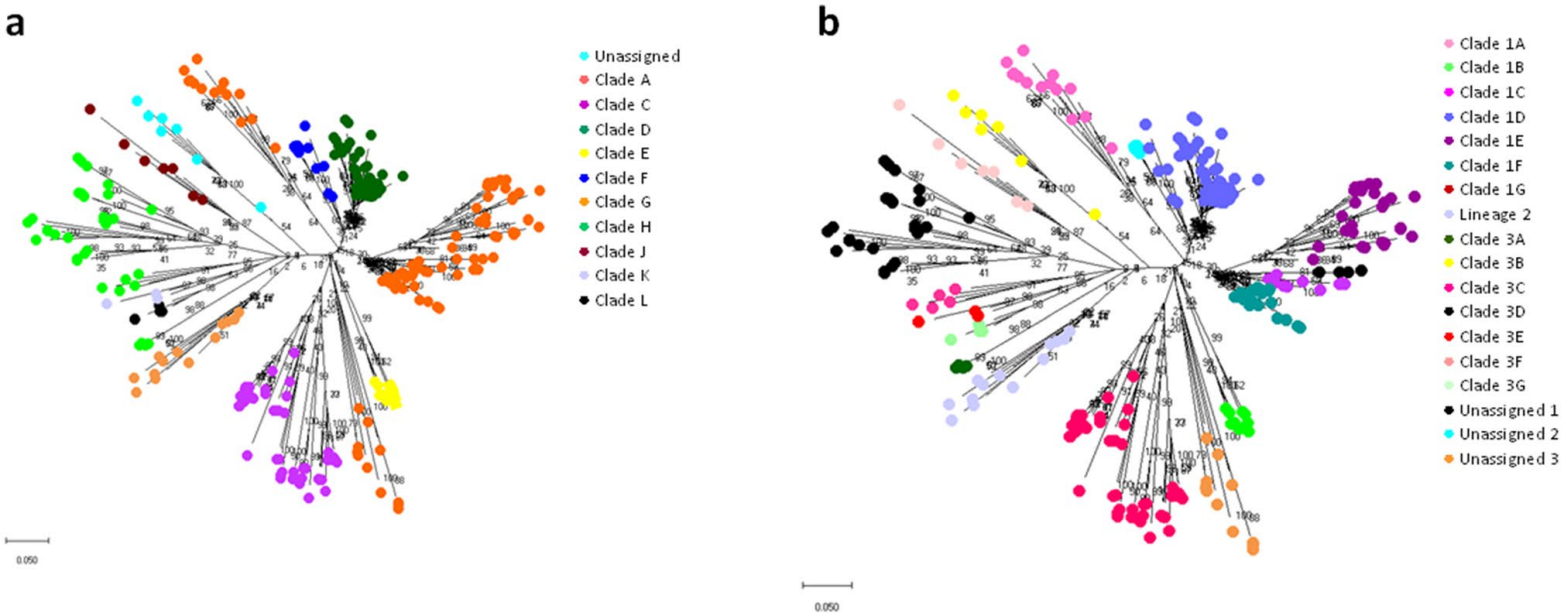
Maximum likelihood phylogenetic representation of 314 PRRSV ORF5 sequences. Taxa are coloured by (**a**) clades^15^ (**b**) lineages^16^.

**Figure 2:**
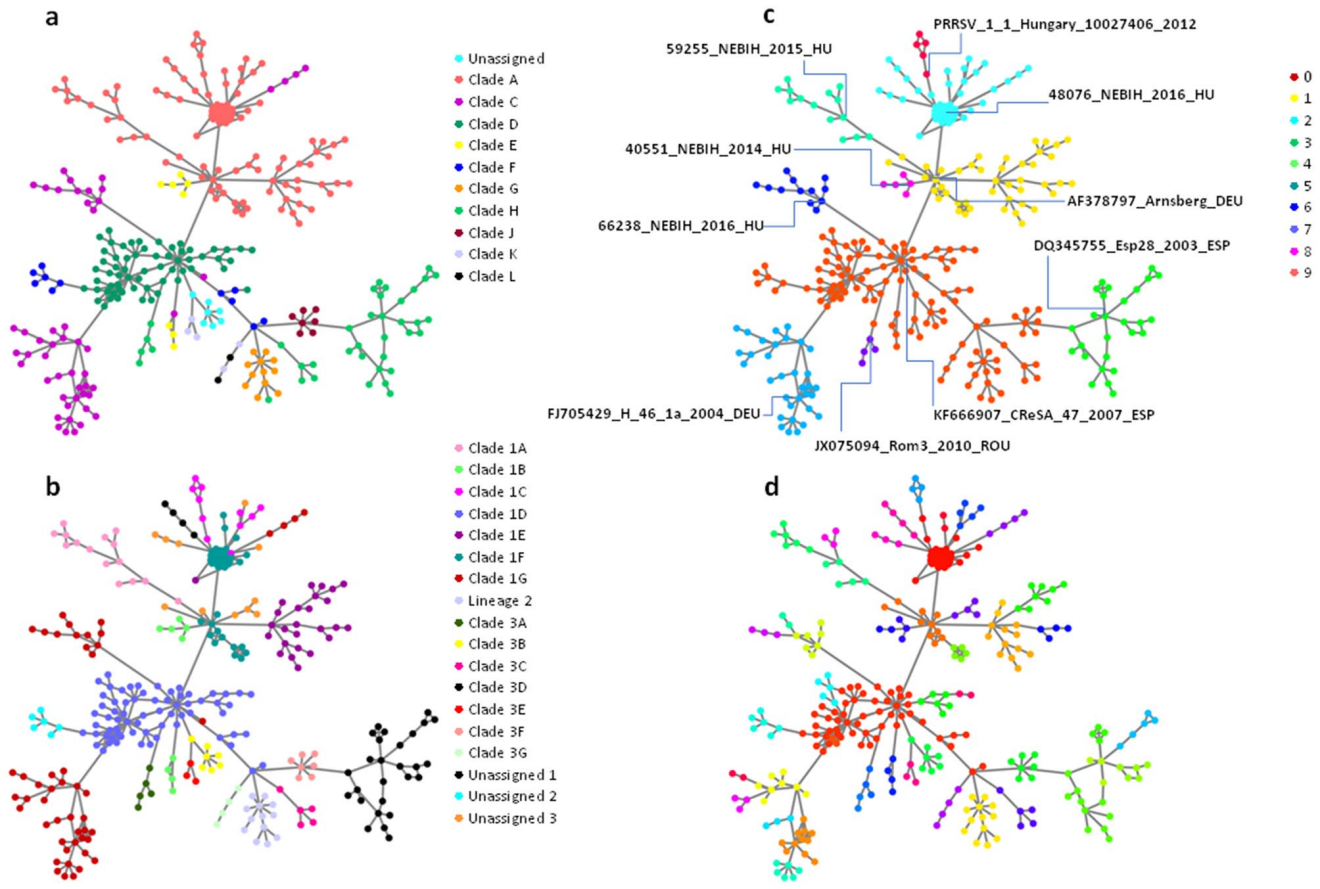
Network representation of 314 PRRSV ORF5 sequences. Nodes are colored by (**a**) clades (**b**) lineages, (**c**) upper and (**d**) lower level of network module hierarchy. Representative sequences of upper level of network module hierarchy are marked.

Maximum likelihood analysis of the 206 Hungarian sequences showed extensive diversity, their average similarity was 86%, and the most distant strains showed only 79% similarity. The Hungarian PRRSV strains belonged to nine clades of the 12^15^ (Fig. 1A) or 11 clades out of 15^16^ (Fig. 1B) within PRRSV1 subtype 1. Clade I (German sequences), Clade K and L (Italian sequences) were absent in Hungary based on^15^, while Clade 3A (Romanian sequences), Clade 3B (Slovenian and Italian sequences), Clade 3E and 3G (Italian sequences) were not present in Hungary according to^16^. The official clades and lineages with high bootstrap values formed distinct clusters both in phylogenetic trees and network visualizations (see ranges of colors in Fig. 1A,B vs. Fig. 2A,B). In contrast, since network visualization is based on centrality metrics, and only the closely related sequences form a distinct cluster, (sub)clades connected with low bootstrap values in the classical phylogenetic tree were not present in the network. For example, subgroups of Clade 1C with 0.24 bootstrap value formed two unrelated distinct lineages in the network (Fig. 2B).

### Network analysis of ORF5 sequences

The pairwise comparison of 314 PRRSV ORF5 sequences generated 97,656 alignment scores (Supplementary Table 1). Our network analysis approach was designed to identify connections between each viral sequence using the highest alignment scores. Due to the continuous nature of these scores, the application of any arbitrary cut-off would introduce a selection bias. By this approach, we identified a connected subnetwork of the viral sequences with 662 edges (Fig. 2A). High alignment scores were observed between the connected sequences along the selected network (Supplementary Figure 1). The distribution of all sequence alignment scores showed a long “right-tail” with three peaks at 85%, 93% and 98%, respectively, which may mark low, medium and high level of similarities between the viral sequences, respectively.

The selected subnetwork diameter (the maximum number of steps between two nodes) was 17. The diameter can be used as a measure of connectivity of a network. The radius (the minimum among the maximum number of steps between a node to all other nodes) was 9, and average number of neighbors was 4.23. It showed scale-free characteristics (power: − 1.302, R-squared: 0.6).

Betweenness and closeness centrality was also calculated for each node. As nodes with high betweenness and closeness centrality scores are more central/closer to the other nodes, they may be the closest available relatives of sources of PRRSV infection in Hungarian herds. Sequence KF666907 CReSA 47 2007 ESP had the highest scores for both metrics in the subnetwork. We also identified an extremely highly connected subnetwork within Clade A, with high similarity scores of 27 nodes including a viral sequence from a modified live vaccine (Porcilis PRRS (MSD)) widely used in Hungary (Fig. 2B).

Highly connected nodes in networks could belong to communities. Several methods were published to identify extensively overlapping network modules. During our analysis, we applied the ModuLand framework^22^. The method generates multiple hierarchical layers of modules, and identifies a representative meta-sequence for each module based on centrality metrics. Phylogenic tree based clade and linage classifications of the selected ORF5 PRRSV sequences were in high concordance with network modules calculated by ModuLand algorithm (Fig. 2C).

The PRRSV ORF5 sequences belonging to the official clades and lineages with high bootstrap values clustered in our network visualizations (see ranges of colors in Fig. 2A,B). By our network analysis, compared with the classical phylogenetic tree of the PRRSV ORF5 sequences, each connection of a certain sequence to the others was much more visible showing the potential genetic relationship among them. Using this approach, the 314 sequences were clustered into 10 modules at the upper hierarchical level (Fig. 2C; Supplementary Table 2), while 49 modules were identified at the lower hierarchical level (Fig. 2D). The algorithm created meta-nodes on the higher hierarchical level that have the highest modular assignment value for the corresponding module at one level below in the hierarchy. These representative sequences selected by the algorithm at the upper level were: KF666907 CReSA 47 2007 ESP, DQ345755 Esp28 2003 ESP, 48,076 NEBIH 2016 HU (identical with Porcilis PRRS (MSD) vaccine strain), AF378797 Arnsberg DEU, JX075094 Rom3 2010 ROU, 59,255 NEBIH 2015 HU, FJ705429 H 46 1a 2004 DEU, 66,238 NEBIH 2016, 40,551 NEBIH 2014 HU, PRRSV1 1 Hungary 10,027,406 2012. These “core” sequences were not identified using the maximum likelihood phylogenetic visualization.

### Practical application of network analysis of ORF5 sequences

**Figure 3:**
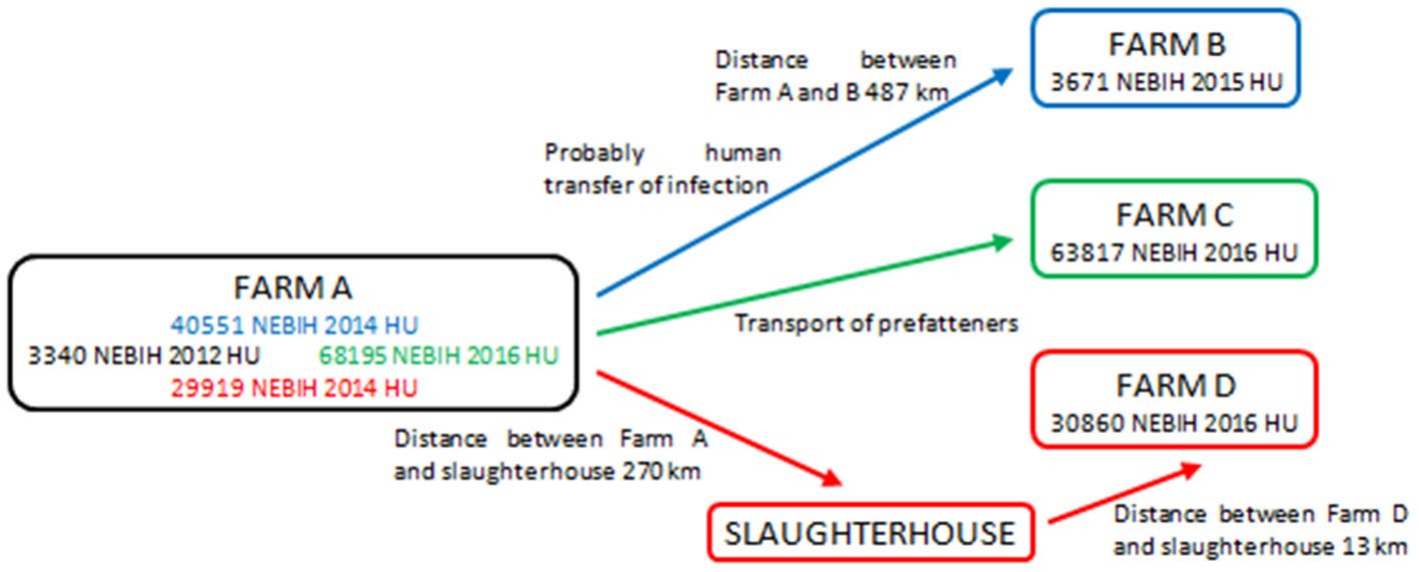
Spreading of PRRSV Clade E strains between farms A, B, C and D in Hungary. Names of the PRRSV sequences identified in the different farms are indicated.

**Figure 4:**
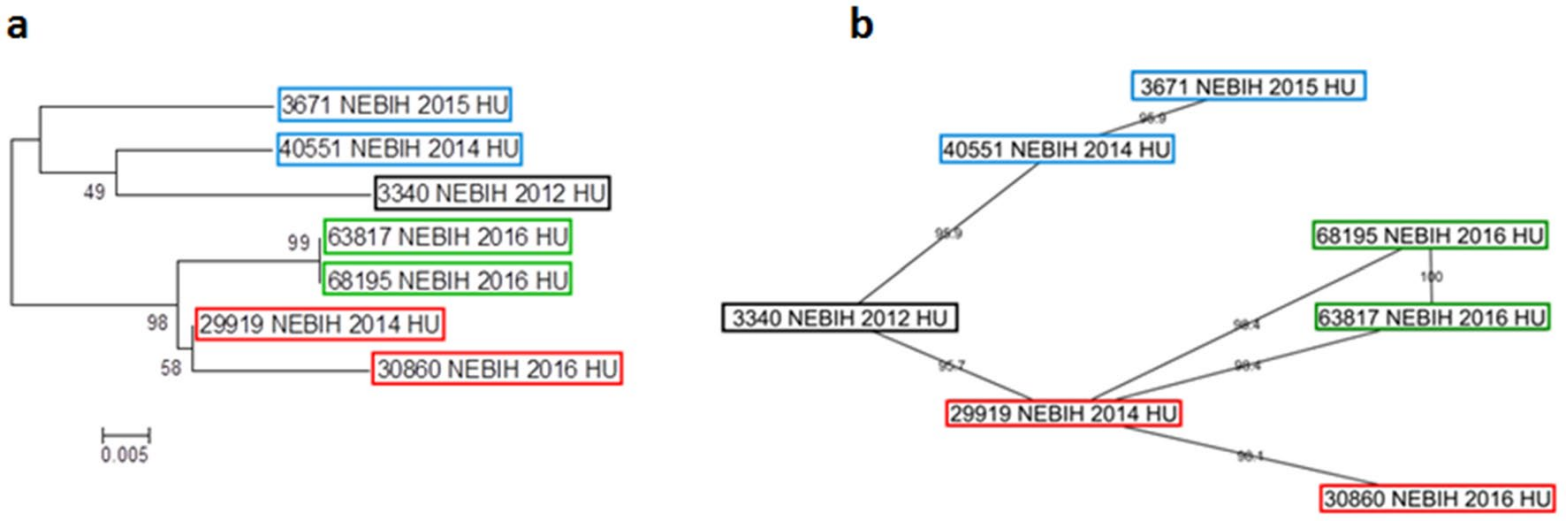
ML phylogenetic (**a**) and network (**b**) analysis of Clade E PRRSV sequences.

Clade E PRRSV (3340 NEBIH 2012 HU) was first found in 2012 in the fattening unit of a PRRSV free multi-site swine farm where breeding animal production, nursery and finishing farm were performed in separate units (Fig. 3, Farm A). PRRSV was most likely introduced into Hungary by infected boars imported from Western Europe, and was detected in the sow herd that provided animals to this fattening unit in 2010. Unfortunately, the sequence of this strain is not available. The virus evolved, and two distinct variants were detected in the breeding farm units of Farm A in 2014 (40,551 NEBIH 2014 HU and 29,919 NEBIH 2014 HU). The changes in the ORF5 gene segments of the PRRSVs were 4.1 and 4.3%, respectively, over the two-year-long period (Fig. 4). Later, a new variant, 29,919 NEBIH 2014 HU was detected in 2016 at this farm (68,195 20 NÉBIH 2016). The change in the ORF5 segment of the PRRSV during this two-year-long period was 1.6%.

In early 2015, 487 km north-east from the above pig farm, a PRRSV outbreak occurred in a farrow-to finish Farm (Fig. 3, Farm B). This PRRSV ORF5 sequence (3671 NEBIH 2015 HU) showed significant (95.9%) similarity with sequence 40551 NEBIH 2014 HU (Fig. 4). There was no swine, swine-origin or feed transport among these farms. Epidemiological investigations revealed that Farm A management visited the PRRSV-free Farm B several times in the pre-infection period, which suggests that PRRSV introduction occurred most likely by human activity. This assumption was further confirmed by the fact that in the proximity of Farm B, no similar sequences were found.

Import of PRRSV infected fatteners posed an enormous risk to the PRRSV eradication in Hungary. Therefore, a PRRSV PCR assay (and subsequent ORF5 sequencing) was compulsory on imported pigs 48 h after their arrival. In these tests, a PRRSV ORF5 sequence (63,817 NEBIH 2016 HU) was detected in a large-scale fattening unit (Fig. 3, Farm C) located 215 km north from Farm A where outplacement of fatteners was performed from without official authorization. 63,817 NEBIH 2016 HU sequence was identical to 68,195 20 NÉBIH 2016, the 2016 PRRSV of Farm A (Fig. 4).

In 2016, a PRRSV outbreak occurred in another pig herd where pig farming was carried out on two separate farms: the production of pre-fattening animals (farrowing and nursery) on one farm, while fattening on the other (Fig. 3, Farm D). The PRRSV identified (30,860 NEBIH 2016 HU) showed 98.1% identity with 29,919 NEBIH 2014 HU previously detected in Farm A (Fig. 4). The distance between Farm A and Farm D is 265 km. The most likely source of contamination is the slaughter of fattening pigs originating from Farm A in a slaughterhouse from which the by-product was taken by the same contractor that also transported pig carcasses from the Farm D farms. At that time, contamination with pig waste could occur, probably due to inadequate disinfection of the transport vehicle. The air-borne transmission of PRRSV from the slaughterhouse to and the Farm D was ruled out based on the 13 km distance.

## Discussion

A crucial element of PRRSV control is revealing the epidemiological link between new outbreaks, and identification of the origin of introduction. In order to achieve this goal, several classical phylogeny methods are applied in eradication programs worldwide. In the phylogenetic tree representation, leaves are sampled pathogens, while internal nodes are most recent common ancestors of the sampled and transmitted pathogens. The drawback of this approach is that if genetic diversity is high, and large number of samples are analyzed, the presence of the large number of internal nodes makes phylogenetic tree representation inconclusive, and difficult to analyze.

Minimum spanning phylogenetic networks overcome these disadvantages by representing the sampled pathogens as nodes, and genetic distances (similarity scores) as edges. The shortcomings of this approach are: (1) every PRRSV sequence will be connected to the network, even if the similarity scores are low. In these cases the algorithm will use the edge with the highest available similarity score and connect the node to the network. These connections will have low alignment scores and most likely do not represent any connections between the two connected sequences, (2) in an MST, each node has one or two connections, and in one network multiple MSTs could exist, (3) if multiple identical PRRSV strains exist, their similarity scores will be the same and their localization in the MST will be interchangeable.

In contrast to previous network algorithms, by using our network analysis, we were able to generate a graph where each node was connected with high alignment scores, without applying a predefined, arbitrary cut-off or computationally extensive algorithms, and connections with low similarity scores were clear outliers and were easy to identify. To avoid random selection of nodes by these caveats, and to make sure that each high similarity score gets presented in the final network, we enriched each node with high alignment score edges, using data from the fully connected network and the result of the Prim`s algorithm. Using this approach, we were able to generate a single similarity network which includes each 314 PRRSV ORF5 sequences in one analysis.

The network analysis of 314 Hungarian and reference PRRSV ORF5 sequences derived from biological samples from swine holdings infected with PRRSV showed that this phylogenetic representation supports the standard clade^15^ or lineage^16^ based classification of individual sequences. Moreover, the nodes representing the sampled PRRSV strains in the different farms were connected and directly, eliminating the possible common ancestor nodes of the classical phylogenetic tree. Enrichment of the number of connections further increased the possible genetic relationship between the between the nodes. These features enabled this method to show the relationships between individual PRRSV sequences in a complex but at the same time easily understandable way. Based on the intense and well documented sampling during the PRRSV Eradication Program, this visualization tool made possible to better identify the potential way of infection among individual pig holdings.

Analysis of the enriched similarity network characteristics revealed additional epidemiological information. Sequences with high betweenness and closeness centrality scores were detected by the ModuLand algorithm^22^. These “core” viruses really played a central role in the origin of PRRSV infections currently occurring in Hungary.

Sequence KF666907 CReSA 47 2007 ESP^23^ (Fig. 2C) represents a PRRSV that was imported into Hungary by an international pig breeding company, and infected almost all the herds of one of the most important pig production counties of the country. This group of viruses, together with a closely related a modified live virus vaccine, are still present in a significant proportion of large breeding farms. An interesting phenomenon of this group is the emergence of farm-specific clusters irrespective of vaccine or wild type virus origin (Fig. 2A,B).

The sequence DQ345755 Esp28 2003 ESP^24^ (Fig. 2C) represents a PRRSV that already appeared in Hungary in 2009, probably via French imported boars but later the largest source of this group was import of fatteners from the Netherlands.

48,076 NEBIH 2016 (Fig. 2C) is 100% identical to the ORF5 sequence of the virus strain in the MLV vaccine of Porcilis PRRS (MSD Animal Health). The above mentioned sequence and the surrounding well defined tight cluster indicate that in flocks where the vaccine was used to control and eradicate the disease, the vaccine virus showed extreme stability. The same phenomenon was not observed in the case of the other MLV vaccines applied in Hungary.

AF378797 Arnsberg DEU^25^ (Fig. 2C) is a German PRRSV strain from the 1990s. From 2001, extensive import of pigs began from Germany, and several herds were infected with these viruses in the same county where DQ345755 Esp28 2003 ESP and its derivates were present. From these original German viruses, a PRRSV clade developed that has only Hungarian members (Clade 1E).

The JX075094 Rom3 2010 ROU^26^ (Fig. 2C) is a sequence originating from Romania. Sequences around it originate also from Romania, indicating that the volume of pig imports from Romania to Hungary is relatively low.

59,255 NEBIH 2015 HU (Fig. 2C) is a virus from a pig farm located in the middle of the Hungarian Great Plain, and the virus was introduced to this farm several years earlier. The first detected member of this group of viruses emerged in 2011 (30,640 NEBIH 2011 HU). Similar viruses were also introduced from 2016 to Hungary with Dutch fatteners and spread to pig farms in the Eastern Hungary region.

The FJ705429 H 46 1a 2004 DEU (Fig. 2C) sequence^25^ is of German origin, that can be the explanation why it appeared in a German-owned pig farm in Hungary (5314 NEBIH 2013 HU).

66,238 NEBIH 2016 is a PRRSV imported from the Netherlands together with other members of this cluster, while PRRSV1 1 Hungary 10,027,406 2012 is a virus of unknown origin that later infected four additional swine herds.

Since these “core sequences” were not identified by classical phylogeny, our data confirm that the central sequences determined by the applied algorithm actually represent starting point along the path of infection and may be suitable for detecting the most likely source and path of infection. On the other hand, our studies revealed the importance of integration of laboratory diagnostics, bioinformatics and epidemiology studies in order to provide an opportunity to precisely determine the spread of PRRSVs.

The results of the practical network analysis of Clade E PRRSV sequences showed that this tool is useful upon infection of individual herds to implement epidemiological investigations (i.e., evaluation of documents available at the farms, identification of slaughterhouses associated with the farms and other facilities (fattening farms) keeping the animals originated from the affected farms). Special attention was paid to official animal transport and animal health documents in relation with the movement of pigs and pig materials (semen, embryo, live breeding, nursery and fattening pigs, slaughterhouse arrival documentation, slaughter logs, animal carcasses, manure, etc.).

The most likely sources of infection and the most feasible way of introduction of the virus into the herd were also evaluated in cooperation with the owners and management of the farms. In order to exclude other possible external contamination events of Farms B-D, the PRRS status of the surrounding pig farms was evaluated by the veterinary authorities. It was checked which feed, from which supplier and by which route was delivered to the farm. It was also verified, which partners could come into contact with the farms regarding the distribution of veterinary medicines, animal nutrition consultancy, rodent pest control and technological maintenance. Movement of persons (personnel, visitors) was also evaluated based on the registers of the farms specified in their official biosecurity manuals. The network visualization (Fig. 4B) fully coincided with the flowchart of the epidemiological investigations (Fig. 3) but these connections were not clearly visible on the classical phylogenetic tree (Fig. 4A).

Determining the sequence distance between the members of clade E it turned out that although it is assumed that 1% of the composition of ORF5 may change every year^12^, much higher genetic distance was found than it was expected based on the time of detection of the different strains. During the PRRSV Eradication Program, we observed in different farms that the introduced virus strain evolved fast in the beginning, and later the evolution slowed down significantly. This phenomenon can be explained on one hand that the introduced PRRSV changed due to vaccine pressure, or on the other hand, PRRSV introduced from a vaccinated farm may have evolved in the other direction in a farm using no or a different vaccine strain. Based on these data, we should be cautious with regard to tightening the potential threshold of % of similarity.

In conclusion, the network based visualization of ORF5 sequence similarities was easy to understand and analyze for private or official veterinarians, even for farm managers during the regular PRRS Eradication Committee meetings in order to evaluate possibilities of the sources of infection of their own farms. This tool was complementary to the classical phylogenic-tree based analysis and data representation, and helped clearly identify potential connections between different viral sequences that support data-driven decisions in the eradication program. However, appropriate sampling is necessary for establishing clear epidemiological links between sequences. If additional sampling had been done in years preceding the official eradication program, results of this approach could have shown different epidemiological interpretation of clade E virus relatedness. By applying network analysis methods, we identified previously unknown epidemiological connections between infected herds, analyzed the virus spreading in time and observed important characteristics of vaccine viruses. In our study, we successfully built and applied network analysis tools in the course of the Hungarian PRRSV Eradication Program.

## Methods

### Sample collection and DNA sequencing

Sequencing of PRRSV strains present in Hungary was performed from biological samples of infected pig farms. This work was carried out occasionally between 2002 and 2013, and 148 sequences were obtained. In contrast, after the initiation of the National PRRSV Eradication Program, sequencing was applied regularly according to the legislation, and 2031 sequences were generated. In order to avoid repetitions, we selected 206 Hungarian sequences that represent 15 out of the 19 counties of the country, the territory with significant swine industry. These sequences were obtained from breeding farms that corresponded to 92% of all breeding sites, as well as 48% of fattening herds in Hungary. In each herd, the index sequence was kept in the analysis. Index sequence was considered the first sequence within each evolutionary variant in the same herd. If a new PRRSV emerged in a farm from external source that did not cluster within the previous farm-specific sequences, and showed more than 2% difference at nucleotide level, this sequence was also included in the phylogenetic studies as a new index sequence.

Mostly serum samples but aborted fetuses and lung tissues from diseased, as well as apparently healthy pigs were tested for the presence of PRRSV with the virotype PRRSV RT-PCR Kit (Qiagen, Hilden, Germany). The complete ORF5 gene from the selected samples was amplified according to previous protocols^27,28^. Sequencing was performed using the Sanger method. The sequences were submitted to GenBank under the accession Numbers (MN102128-MN102334). In order to classify and to find the possible origin of the Hungarian PRRSV strains, 108 reference PRRSV sequences systematically selected from^16^ and GenBank were also involved into the phylogenetic analyses.

### Phylogenetic analysis of ORF5 sequences

Phylogenetic analysis was performed by the Molecular Evolutionary Genetics Analysis version 6.0 (MEGA 6.0) software^29^. After best fit model selection (Supplementary Table 3), the maximum likelihood algorithm was used with General Time Reversible model (GTR) and four categories of Gamma-distributed with Invariant sites (G + I). The topology of the phylogenetic tree was confirmed with 1000 bootstrap replicates.

### Network analysis of ORF5 sequences

ORF5 sequence similarity networks were created by applying pairwise comparison between each sequence involved in the analysis. Sequence alignment scores were calculated by Needleman–Wunsch and Smith–Waterman algorithms for global and local pairwise sequence alignments. For each comparison, the alignment was performed twice by switching the reference and subject sequences. From the two alignment scores, the higher was used for subsequent analysis.

The results of the pairwise comparisons were stored in an N × N matrix where each column and row represented a sequence, and the values were the alignment scores. This matrix was used to create a fully connected, edge weighted network where each node represented a PRRSV ORF5 sequence, while the edges represented their potential connections, and the edge weights (similarity values) were the calculated alignment scores.

**Figure 5:**
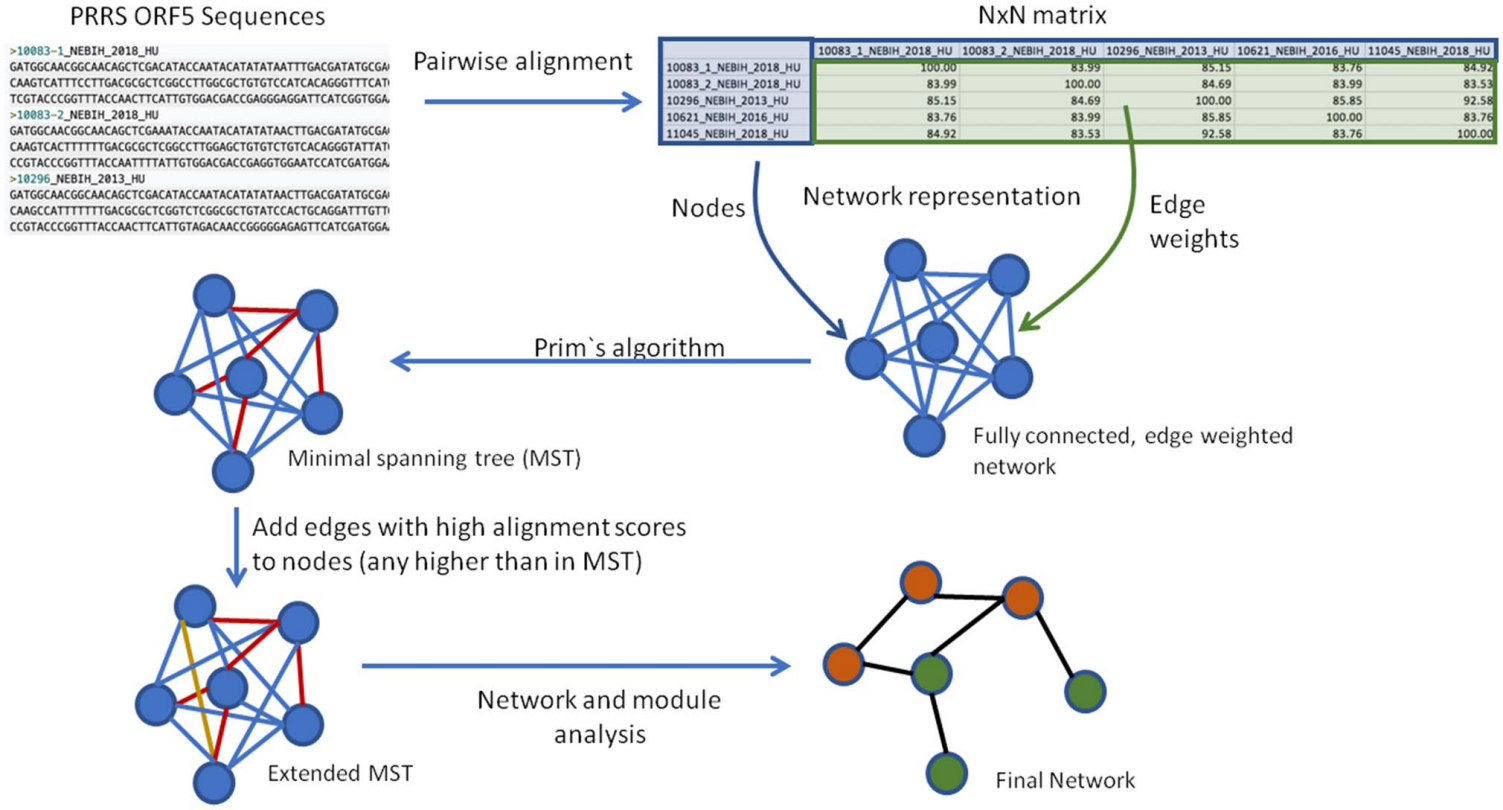
Schematic overview of generating enriched minimum spanning network (similarity network) of 314 PRRSV sequences.

To generate a network where each viral sequence is a member, and their connections are represented with high alignment scores, the Prim`s algorithm was applied in order to identify a minimum spanning tree (MST). At the first step we transformed each weight (100-weight) in the full network to make high scores low and low scores high. Next the Prim`s algorithm applied to select a MST. In a spanning tree every node has only one or two connections which might be insufficient to represent connections between viral genomes. For example, if multiple edges have the same minimum alignment scores, the algorithm will randomly pick one and not select all connections with the same high weights. In our analysis, we wanted to assure that each of these edges is represented in the final network. After an initial MST was identified, we enriched the graph with additional edges by adding every edge for each node which had equal or higher weight than the edges in the initial MST to the corresponding node to create the extended MST (Fig. 5). All data analyses were performed with R 3.4.1 for Linux.

Network analysis was performed on the extended MST with Cytoscape 3.6.0. Network diameter, radius, average number of neighbors and values to determinate scale-free characteristics were calculated using NetworkAnalyzer tool. For visualization purposes Allegro Spring-Electric layout with edge weights were applied using AllergoLayout. Network modules were identified by ModuLand framework^22^, and edge weights were used for modularization process.

### Practical application of network analysis of ORF5 sequences

In order to demonstrate the practical applicability of the network analysis of ORF5 sequences, we chose PRRSV clade E which was very rarely found in Hungary, and originated from a well-defined herd. The index sequence of clade E was identified in 2012 in a large-scale pig farm (Farm A). When we identified similar PRRSV ORF5 sequences in different geographical location using the network analysis tool, we traced PRRSV introduction by animal health management investigation methods using the national registers of farm and animal transport data, as well as local veterinary administration and farm management records. We also attempted to detect epidemiological relationships between the original PRRSV source and other Clade E PRRSVs detected in the period of 2012–2017.

## Supplementary information

Supplementary Legends.

Supplementary Figure 1.

Supplementary Table 1.

Supplementary Table 2.

Supplementary Table 3.

## Data Availability

The datasets generated during and/or analyzed during the current study are available from the corresponding author on reasonable request.
