# Supplementary material for: Investigations on spreading of PRRSV among swine herds by improved minimum spanning network analysis
Source: Sci Rep. 2020 Nov 5;10:19217. doi: 10.1038/s41598-020-75516-5 (PMC7645787; doi:10.1038/s41598-020-75516-5)
Supplement: Supplementary file 1 — Supplementary Legends. [file 41598_2020_75516_MOESM1_ESM.docx]

**Supplementary Figure 1.** Histogram and density plots of 662 selected alignment scores and 97656 alignment scores from all pairwise comparisons between 314 sequences.

**Supplementary Table 1.** Alignment scores generated by pairwise comparison of 314 PRRSV ORF5 sequences

**Supplementary Table 2.** Clustering of 314 PRRRSV ORF5 sequences into 49 modules at the lower hierarchical level and into 10 modules at the upper hierarchical level using ModuLand algorithm.

**Supplementary Table 3.**

Best fit model selection of 314 PRRSV sequences for generating maximum likelihood phylogenetic tree using the MEGA6 software
