# Supplementary figures and images for: Investigations on spreading of PRRSV among swine herds by improved minimum spanning network analysis
Source: Sci Rep. 2020 Nov 5;10:19217. doi: 10.1038/s41598-020-75516-5 (PMC7645787; doi:10.1038/s41598-020-75516-5)

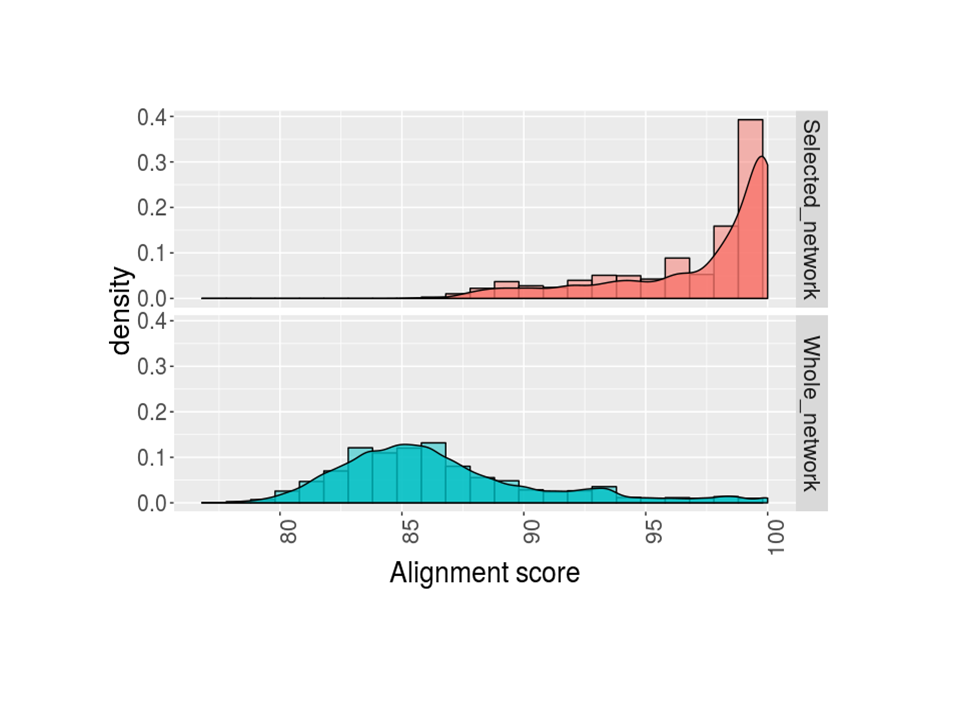

Supplement: Supplementary file 2 — Supplementary Figure 1. [file 41598_2020_75516_MOESM2_ESM.tif]
